# Supplementary material for: Hierarchical ground-state crystals underlying Hertzian quasicrystals
Source: Nat Commun. 2025 Nov 25;16:11269. doi: 10.1038/s41467-025-66158-0 (PMC12717090; doi:10.1038/s41467-025-66158-0)
Supplement: Supplementary file 1 — Supplementary Information [file 41467_2025_66158_MOESM1_ESM.pdf]

Supplementary Information: Hierarchical ground-state crystals underlying Hertzian Quasicrystals

Yao Li[1],* Yiwei Wang[1], Yingke Geng[1], Wenyu Liu[1], Fangfu Ye[2,3],† and Jeff Z. Y. Chen[4‡]
[1] *School of Physics and Key Laboratory of Functional Polymer Materials of Ministry of Education, Nankai University, and Collaborative Innovation Center of Chemical Science and Engineering, Tianjin 300071, China.*
[2] *Beijing National Laboratory for Condensed Matter Physics and Laboratory of Soft Matter Physics, Institute of Physics, Chinese Academy of Sciences; Beijing 100190, China.*
[3] *Oujiang Laboratory (Zhejiang Lab for Regenerative Medicine, Vision, and Brain Health), Wenzhou Institute, University of Chinese Academy of Sciences, Wenzhou, Zhejiang 325000, China. and*
[4]*Department of Physics and Astronomy, University of Waterloo; Waterloo, Ontario, N2L 3G1, Canada.*

MINIMIZATION OF LATTICE ENERGIES

In this SM, the procedure that we used to obtain the energies of the pentagan-related lattices is outline. The aim is to minimize the total reduced energy per particle

$$\frac{U}{\varepsilon} = \frac{1}{2N}\sum_{i=1}^{N}\sum_{j} u(r_{ij}) \tag{1}$$

where N is the number of particles in the primitive unit cell, and j runs through all particles within the force range σ in the Hertzian potential.

Cairo Lattice. In this case, only one parameter, θ seen in Fig. S1, is needed to specify all particles. The two used lattice vectors are,

$$\mathbf{a}_1 = L\left(\sin\theta - \cos\theta + 1, \sin\theta + \cos\theta - 1\right) \tag{2}$$

$$\mathbf{a}_2 = L\left(1 - \sin\theta - \cos\theta, \sin\theta - \cos\theta + 1\right) \tag{3}$$

where $L = \frac{1}{2}\sqrt{3}\csc\frac{\theta}{2}/\sqrt{\rho^*}$.

There are 6 particles in a primitive cell of the Cairo lattice, shown in Fig. S1. Their coordinates can be specified by

$$\begin{pmatrix} \mathbf{r}_1 \\ \mathbf{r}_2 \\ \mathbf{r}_3 \\ \mathbf{r}_4 \\ \mathbf{r}_5 \\ \mathbf{r}_6 \end{pmatrix} = L\begin{pmatrix} 0 & 0 \\ 1 & 0 \\ 1-\cos\theta-\sin\theta & \sin\theta-\cos\theta \\ 1-\cos\theta & \sin\theta \\ 0 & 1 \\ \sin\theta-\cos\theta & \sin\theta+\cos\theta-1 \end{pmatrix} \tag{4}$$

The energy shown in Fig. 2 of the main text is the result of the minimization with respect to θ.

Chengtu Lattice. The Chengtu lattice can be specified by a single parameter θ as well, as shown in Fig. S2. The two lattice vectors are,

$$\mathbf{a}_1 = L\left(3\sin\frac{\theta}{2}\ ,\ \sqrt{3}\sin\frac{\theta}{2}\right) \tag{5}$$

$$\mathbf{a}_2 = L\left(0\ ,\ 2\sqrt{3}\sin\frac{\theta}{2}\right) \tag{6}$$

where $L = 3^{-3/4}\sqrt{5/2\rho^*}\csc(\theta/2)$.

There are 5 particles in a primitive cell of the Chengtu lattice, shown in Fig. S2. The coordinates of these can be

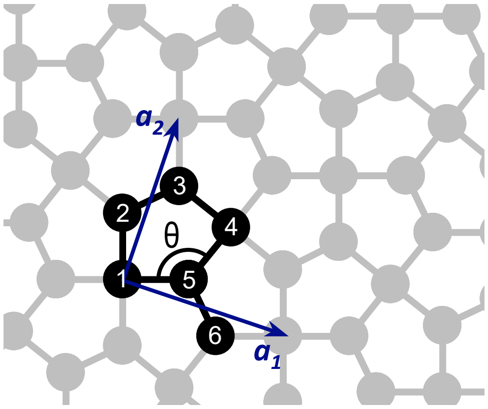

FIG. S1. Primitive unit cell of a Cairo Lattice. The $N = 6$ particles considered are labeled inside the circles.

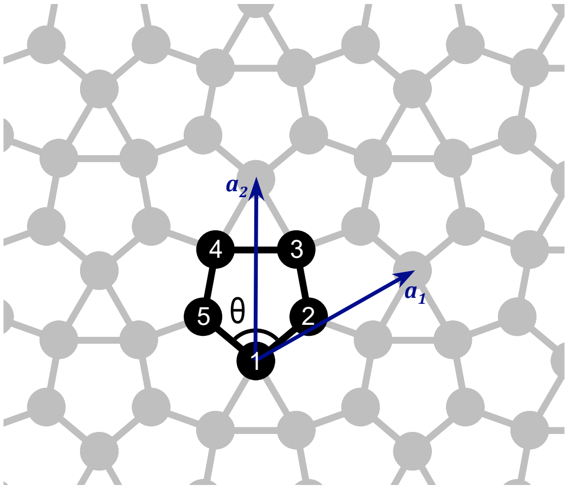

FIG. S2. Primitive unit cell of a Chengtu Lattice. The $N = 5$ particles considered are labeled inside the circles.

specified by,

$$\begin{pmatrix} \mathbf{r}_1 \\ \mathbf{r}_2 \\ \mathbf{r}_3 \\ \mathbf{r}_4 \\ \mathbf{r}_5 \end{pmatrix} = L \begin{pmatrix} 0 & 0 \\ \sin\frac{\theta}{2} & \cos\frac{\theta}{2} \\ \sqrt{3}\sin[(3\theta-\pi)/6] & \sqrt{3}\cos[(3\theta-\pi)/6] \\ \sqrt{3}\sin[(\pi-3\theta)/6] & \sqrt{3}\cos[(3\theta-\pi)/6] \\ -\sin\frac{\theta}{2} & \cos\frac{\theta}{2} \end{pmatrix} \tag{7}$$

The energy shown in Fig. 2 of the main text is the result of minimization with respect to θ.

PenHex-Str Lattice. The PenHex-Str lattice can be specified by two parameters, θ and γ, as shown in Fig. S3. The two lattice vectors are,

$$\mathbf{a}_1 = L\left(4\sin\frac{\theta}{2} + 2\cos\gamma, 0\right) \tag{8}$$

$$\mathbf{a}_2 = L\left(2\sin\frac{\theta}{2} + \cos\gamma, 2\cos\frac{\theta}{2} + \sin\gamma\right) \tag{9}$$

where $L = \sqrt{5}\rho^{*-1/2}\left[4\cos(\theta/2-\gamma) + 4\sin\theta + \sin 2\gamma\right]^{-1/2}$.

There are 5 particles in a primitive cell of PenHex-Str lattice, shown in Fig. S3. The coordinates can be specified

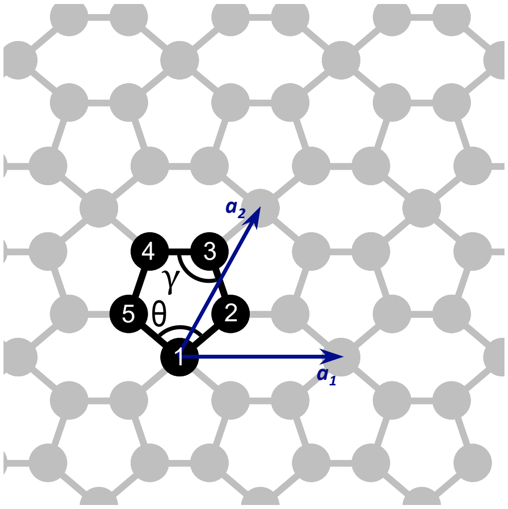

FIG. S3. Primitive unit cell of a PenHex-Str Lattice. The $N = 5$ particles considered are labeled inside the circles.

by,

$$\begin{pmatrix} \mathbf{r}_1 \\ \mathbf{r}_2 \\ \mathbf{r}_3 \\ \mathbf{r}_4 \\ \mathbf{r}_5 \end{pmatrix} = L \begin{pmatrix} 0 & 0 \\ \sin\frac{\theta}{2} & \cos\frac{\theta}{2} \\ \sin\frac{\theta}{2} + \cos\gamma & \cos\frac{\theta}{2} + \sin\gamma \\ -\sin\frac{\theta}{2} & \cos\frac{\theta}{2} \\ -\sin\frac{\theta}{2} - \cos\gamma & \cos\frac{\theta}{2} + \sin\gamma \end{pmatrix} \quad (10)$$

The energy shown in Fig. 2 of the main text is the result of minimization with respect to θ and γ.

PenTri-Rec Lattice. The PenTri-Rec lattice can be specified by ten parameters, which are k, x_2, y_2, y_3, x_6, y_6, x_7, y_7, y_8 and x_9. as shown in Fig. S4. The parameters x_i and y_i denote the spatial coordinates of the ith particle, The parameter k is defined as the magnitude ratio of the two lattice vectors,

$$\mathbf{a}_1 = L\,(\ 1\ ,\ 0\ ) \quad (11)$$
$$\mathbf{a}_2 = L\,(\ 0\ ,\ k\ ) \quad (12)$$

where $L = \sqrt{16/(\rho^* k)}$.

There are 16 particles in a primitive cell of PenTri-Rec lattice, shown in Fig. S4. The coordinates can be specified by,

$$\begin{pmatrix} \mathbf{r}_1 \\ \mathbf{r}_2 \\ \mathbf{r}_3 \\ \mathbf{r}_4 \\ \mathbf{r}_5 \\ \mathbf{r}_6 \\ \mathbf{r}_7 \\ \mathbf{r}_8 \\ \mathbf{r}_9 \\ \mathbf{r}_{10} \\ \mathbf{r}_{11} \\ \mathbf{r}_{12} \\ \mathbf{r}_{13} \\ \mathbf{r}_{14} \\ \mathbf{r}_{15} \\ \mathbf{r}_{16} \end{pmatrix} = L \begin{pmatrix} 0 & 0 \\ x_2 & y_2 \\ \frac{1}{4} & y_3 \\ \frac{1}{2} - x_2 & 2y_3 - y_2 \\ \frac{1}{2} & 2y_3 \\ x_6 & y_6 \\ x_7 & y_7 \\ \frac{1}{2} - x_7 & y_8 \\ x_9 & 2y_3 - y_6 - k \\ -x_2 & y_2 \\ -\frac{1}{4} & y_3 \\ -x_6 & y_6 \\ -x_7 & y_7 \\ -\frac{1}{2} + x_7 & y_8 \\ \frac{1}{2} + x_2 & 2y_3 - y_2 \\ 1 - x_9 & 2y_3 - y_6 - k \end{pmatrix} \quad (13)$$

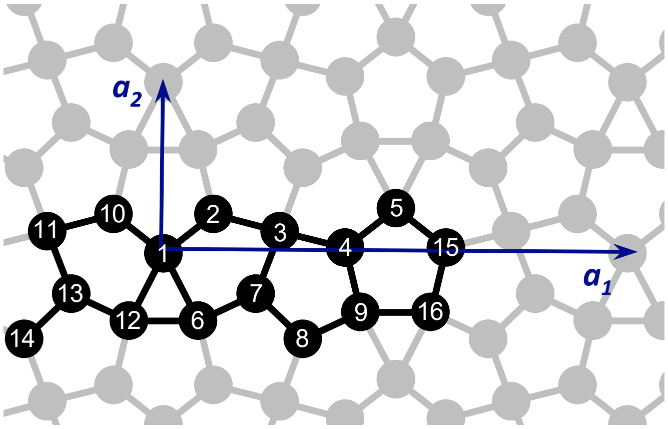

FIG. S4. Primitive unit cell of a PenTri-Rec Lattice. The $N = 16$ particles considered are labeled inside the circles.

The energy shown in Fig. 2 of the main text is the result of minimization with respect to k, x_2, y_2, y_3, x_6, y_6, x_7, y_7, y_8, and x_9.

Agra Lattice. The Agra lattice can be specified by five parameters, which are x_2, y_2, x_3, y_3, and x_4, as shown in Fig. S5. The parameters x_i and y_i denote the spatial coordinates of the ith particle. The two lattice vectors are,

$$\mathbf{a}_1 = L\left(\frac{1}{\sqrt{2}}, \frac{1}{\sqrt{2}}\right) \tag{14}$$

$$\mathbf{a}_2 = L\left(-\frac{1}{\sqrt{2}}, \frac{1}{\sqrt{2}}\right) \tag{15}$$

where $L = \sqrt{22/\rho^*}$.

There are 22 particles in a primitive cell of Agra lattice, shown in Fig. S5. The coordinates can be specified by,

$$\begin{pmatrix} \mathbf{r}_1 \\ \mathbf{r}_2 \\ \mathbf{r}_3 \\ \mathbf{r}_4 \\ \mathbf{r}_5 \\ \mathbf{r}_6 \\ \mathbf{r}_7 \\ \mathbf{r}_8 \\ \mathbf{r}_9 \\ \mathbf{r}_{10} \\ \mathbf{r}_{11} \\ \mathbf{r}_{12} \\ \mathbf{r}_{13} \\ \mathbf{r}_{14} \\ \mathbf{r}_{15} \\ \mathbf{r}_{16} \\ \mathbf{r}_{17} \\ \mathbf{r}_{18} \\ \mathbf{r}_{19} \\ \mathbf{r}_{20} \\ \mathbf{r}_{21} \\ \mathbf{r}_{22} \end{pmatrix} = L \begin{pmatrix} 0 & \frac{1}{\sqrt{2}} - x_4 \\ x_2 & y_2 \\ x_3 & y_3 \\ x_4 & 0 \\ \frac{1}{\sqrt{2}} - y_2 & -x_2 \\ \frac{1}{\sqrt{2}} - y_3 & -x_3 \\ \frac{\sqrt{2}}{4} & -\frac{\sqrt{2}}{4} \\ 0 & -\frac{1}{\sqrt{2}} + x_4 \\ x_2 & -y_2 \\ x_3 & -y_3 \\ -\frac{1}{\sqrt{2}} + y_2 & -x_2 \\ -\frac{1}{\sqrt{2}} + y_3 & -x_3 \\ -\frac{\sqrt{2}}{4} & -\frac{\sqrt{2}}{4} \\ -x_2 & y_2 \\ -x_3 & y_3 \\ -x_4 & 0 \\ -x_3 & -y_3 \\ -x_2 & -y_2 \\ -y_2 & x_2 - \frac{1}{\sqrt{2}} \\ -y_3 & x_3 - \frac{1}{\sqrt{2}} \\ y_2 & x_2 - \frac{1}{\sqrt{2}} \\ y_3 & x_3 - \frac{1}{\sqrt{2}} \end{pmatrix} \tag{16}$$

The energy shown in Fig. 2 of the main text is the result of minimization with respect to x_2, y_2, x_3, y_3, and x_4.

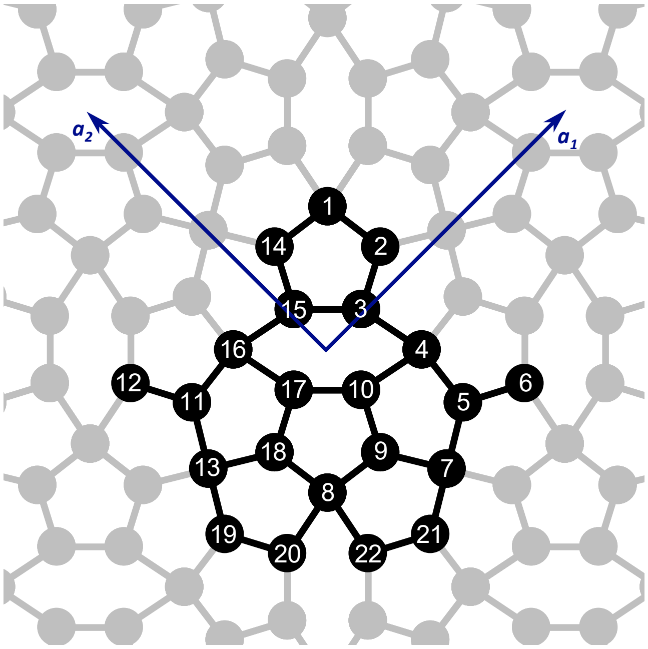

FIG. S5. Primitive unit cell of a Agra Lattice. The $N = 22$ particles considered are labeled inside the circles.

DOUBLE-TANGENT CONSTRUCTION METHOD TO DETERMINE COEXISTENCE

The two branches of the mean energies per unit area, analytically obtained for two candidate structures, 1 and 2 respectively, are used for the double-tangent construction. The coexistence densities are determined by solving the following conditions for ρ_1^* and ρ_2^*,

$$E_1'(\rho_1^*) = E_2'(\rho_2^*), \tag{17}$$

and

$$E_1(\rho_1^*) - E_1'(\rho_1^*)\rho_1^* = E_2(\rho_2^*) - E_2'(\rho_2^*)\rho_2^*. \tag{18}$$

Here the symbol $'$ represents the first derivative of the energy branch, and ρ_1^* and ρ_2^* are the two reduced densities. The mean energy per area is defined by

$$E(\rho^*) = \rho^* U(\rho^*)$$

where $U(\rho^*)$ is the energy per particle defined in (1) above.

* liyao@nankai.edu.cn
† fye@iphy.ac.cn
‡ jeffchen@uwaterloo.ca
